# Ceftriaxone as a Novel Therapeutic Agent for Hyperglutamatergic States: Bridging the Gap Between Preclinical Results and Clinical Translation

**DOI:** 10.3389/fnins.2022.841036

**Published:** 2022-07-05

**Authors:** Osama A. Abulseoud, Fawaz Alasmari, Abdelaziz M. Hussein, Youssef Sari

**Affiliations:** ^1^Department of Psychiatry and Psychology, Alex School of Medicine at Mayo Clinic, Phoenix, AZ, United States; ^2^Department of Pharmacology and Experimental Therapeutics, University of Toledo, Toledo, OH, United States; ^3^Department of Pharmacology and Toxicology, College of Pharmacy, King Saud University, Riyadh, Saudi Arabia; ^4^Department of Medical Physiology, Faculty of Medicine, Mansoura University, Mansoura, Egypt

**Keywords:** ceftriaxone, glutamate, GLT-1, neurological disorder, psychiatric disorder

## Abstract

Dysregulation of glutamate homeostasis is a well-established core feature of neuropsychiatric disorders. Extracellular glutamate concentration is regulated by glutamate transporter 1 (GLT-1). The discovery of a beta-lactam antibiotic, ceftriaxone (CEF), as a safe compound with unique ability to upregulate GLT-1 sparked the interest in testing its efficacy as a novel therapeutic agent in animal models of neuropsychiatric disorders with hyperglutamatergic states. Indeed, more than 100 preclinical studies have shown the efficacy of CEF in attenuating the behavioral manifestations of various hyperglutamatergic brain disorders such as ischemic stroke, amyotrophic lateral sclerosis (ALS), seizure, Huntington’s disease, and various aspects of drug use disorders. However, despite rich and promising preclinical data, only one large-scale clinical trial testing the efficacy of CEF in patients with ALS is reported. Unfortunately, in that study, there was no significant difference in survival between placebo- and CEF-treated patients. In this review, we discussed the translational potential of preclinical efficacy of CEF based on four different parameters: (1) initiation of CEF treatment in relation to induction of the hyperglutamatergic state, (2) onset of response in preclinical models in relation to onset of GLT-1 upregulation, (3) mechanisms of action of CEF on GLT-1 expression and function, and (4) non-GLT-1-mediated mechanisms for CEF. Our detailed review of the literature brings new insights into underlying molecular mechanisms correlating the preclinical efficacy of CEF. We concluded here that CEF may be clinically effective in selected cases in acute and transient hyperglutamatergic states such as early drug withdrawal conditions.

## Introduction

Glutamate dysregulation is evident in preclinical models of neurological and psychiatric disorders (Tsai et al., 1998; Campos et al., 2012; Stephens et al., 2014; Miladinovic et al., 2015). Increased extracellular glutamate concentrations were observed in animals exposed to ethanol (Das et al., 2015), ischemia (Ueda et al., 1992), and amyotrophic lateral sclerosis (ALS) (Andreassen et al., 2001). There are several possible mechanisms involving the dysregulation of glutamate homeostasis in neurological and psychiatric disorders. For instance, nicotine exposure increased extracellular glutamate concentrations by stimulation of nicotinic acetylcholine receptors (nAChRs) (Konradsson-Geuken et al., 2009). This increase in glutamate concentrations might be associated with alterations in glutamate receptors and transporters in pre- and post-synaptic neurons as well as in astrocytes. Chronic ethanol consumption reduced the expression of major glutamate transporters such as glutamate transporter-1 (GLT-1 whose human homolog is excitatory amino acid transporter 2, EAAT2), and increased extracellular glutamate concentrations in the nucleus accumbens (NAc) of male alcohol-preferring (P) rats (Das et al., 2015). Increase in glutamate efflux from pre-synaptic neurons (Nishizawa, 2001) and reversal of GLT-1 (Roettger and Lipton, 1996; Seki et al., 1999) caused excitotoxicity in an ischemic stroke model. It has been found that global ischemia increased extracellular glutamate concentrations by inhibition of the nitric oxide pathway in rats (Zhang et al., 1993).

It is important to note that the majority of extracellular glutamate concentration is regulated mainly by GLT-1 (Danbolt, 2001). Thus, finding an upregulator of this transporter would be clinically relevant. Indeed, Rothstein et al. screened more than 1,000 FDA-approved compounds (Rothstein et al., 2005) and found that mice treated with beta (β-)-lactam antibiotics, specifically ceftriaxone (CEF), for 5 days increased the transcription level of GLT-1 gene, which led to the upregulation of GLT-1 protein. This effect was observed 48 h after drug administration and persisted for at least 7 days. Furthermore, CEF injection at 200 mg/kg/day (IP) for 5-7 days increased GLT-1 expression and glutamate uptake in an animal model of Parkinson’s disease, and this effect was observed for 4 and 7-14 days, respectively, after treatments in naïve rats (Chotibut et al., 2014). In addition, the IP injection of CEF (200 mg/kg) for 5-7 days upregulated GLT-1 in the hippocampus and spinal cord, and this effect was observed 3 months after the last CEF IP injection (Rothstein et al., 2005). The results of these studies suggest that repeated CEF administration at adequate dose upregulates GLT-1 in certain brain regions as early as 2 days, and that the GLT-1 upregulation lasts for an extended period of time up to 3 months.

Ceftriaxone has been reported extensively to attenuate neurological and psychiatric manifestations in part by upregulation of astrocytic glutamate transporters, including GLT-1 and cystine/glutamate antiporter (xCT), in mesocorticolimbic brain regions (Sari et al., 2011; Rao et al., 2015b; Zumkehr et al., 2015; Guan et al., 2016; Krzyżanowska et al., 2017). It is important to note that there are at least two GLT-1 isoforms, GLT-1a and GLT-1b (Berger et al., 2005). We reported in a previous study from Sari’s laboratory that CEF treatment upregulated both GLT-1a and GLT-1b in the prefrontal cortex and nucleus accumbens (Alhaddad et al., 2014a). It is well-known that GLT-1b is mainly expressed in astrocytes, and that GLT-1a is predominantly located in astrocytes and neurons (Berger et al., 2005; Holmseth et al., 2009). Although, the majority of GLT-1 is expressed in astrocytes, which are upregulated with CEF treatment, we believe that upregulation of neuronal GLT-1 by CEF treatment also plays a critical role in regulating extracellular glutamate or hyperglutamatergic state. As such, studies are warranted to translate successful preclinical results of CEF into proof-of-concept pilot studies. It is also warranted to investigate the correlation between the induction time of hyperglutamatergic state associated with neuropsychiatric disorders and appropriate time for initiating therapy. Furthermore, investigation of the onset of action of this compound in upregulating GLT-1 and xCT expression is important to establish a strategical plan for the duration of treatment. In addition to activation of GLT-1 and xCT, studies from Sari’s laboratory also showed that CEF’s attenuation of ethanol-seeking behavior is driven by upregulated equilibrative nucleoside transporter type-1 (ENT-1) (Sari et al., 2013b) and water channel aquaporin-4 (AQP-4) (Lee et al., 2013). In addition, studies have shown that CEF induced neuroprotection and attenuated proinflammatory cytokines in a rat model of traumatic brain injury (Wei et al., 2012; Zhao et al., 2020) and neuropathic pain (Amin et al., 2012).

We argue here that the translational block may stem from the fact that in the majority of these studies, CEF was administered prior to the induction of acute hyperglutamatergic state and, hence, prior to the onset of glutamate-induced excitotoxicity. This “prophylactic approach” made it possible for CEF to offset the increase of synaptic glutamate as soon as it takes place. This scenario is quite different from initiating CEF treatment in humans with refractory psychiatric or neurological diseases where chronic hyperglutamatergic states have already caused neuronal toxicity, and neuronal damage may not benefit from upregulation of glutamate transporter. In this review, we discussed the potential therapeutic effects of CEF, administered prior to or post induction of neurological disorders, on attenuating the dysregulated glutamatergic system of preclinical models of neurological and psychiatric disorders. We also discussed here the applicability of translating the pre-clinical data into humans and characteristics of using CEF in proof-of-concept clinical trials to test its efficacy in attenuating manifestations of certain neuropsychiatric disorders.

## Glutamatergic System Under Physiological Conditions

Glutamate is a major excitatory neurotransmitter in the central nervous system. It serves as an important intermediate in energy metabolism (Massucci et al., 2013), and it is a precursor for gamma-aminobutyric acid (GABA) and glutamine (White, 1981; Hertz, 2013). Glutamate involves the glutamate/cystine antiporter system to regulate glutathione production (Shih et al., 2006). Moreover, energy production was found to be regulated by glutamate by metabolism of amino acids to carbohydrates through tricarboxylic acid (TCA) cycle (Schousboe et al., 2014). It is important to note that neuronal glutamate is synthesized primarily from either glutamine (by glutaminase) or from α-ketoglutarate (by glutamate dehydrogenase) (Onetti et al., 2009; Rowley et al., 2012). Vesicular glutamate transporter (vGLUT) uptakes the synthesized glutamate into vesicles in pre-synaptic glutamatergic neurons, and the packaged glutamate is released upon stimulation into the synaptic cleft (Benarroch, 2010). Glutamate can bind to and activate a variety of metabotropic (mGluRs) and ionotropic glutamate receptors (iGluRs) (Niswender and Conn, 2010; Traynelis et al., 2010). The α-amino-3-hydroxy-5-methylisoxazole-4-propionic acid (AMPA) receptor, N-methyl-d-aspartate (NMDA) receptor, and kainic acid receptors are iGluRs located mainly in post-synaptic neurons. Studies reported that extracellular Glu uptake is mediated through the family of excitatory amino acid transporters (EAATs) expressed mainly in astrocytes (Danbolt, 2001) and, to a lesser extent, in neurons (Rothstein et al., 1994). Importantly, high synaptic glutamate concentrations can lead to excitotoxicity, and it is essential to keep glutamate concentration below the toxic level by modulating EAATs (Liang et al., 2008).

**FIGURE 1:**
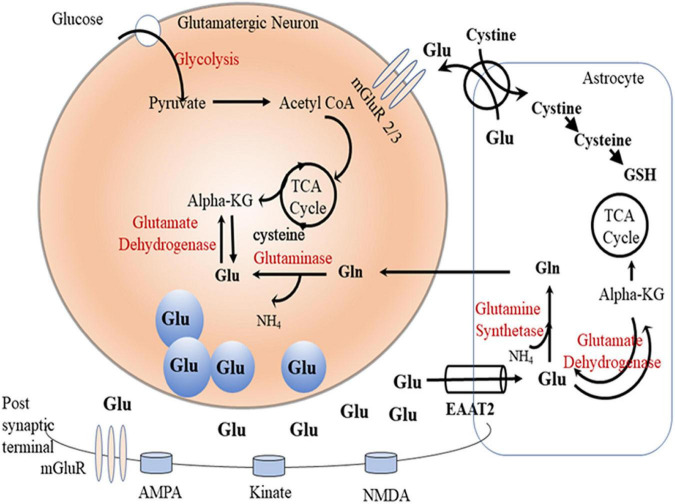
Glutamate-glutamine cycle. Glutamate (Glu) is released from presynpatic glutamatergic neuronal vesicles to the synaptic cleft where it exerts its action by transient binding to glutamatergic receptors (AMPS, kinate, and NMDA). Dissociated glutamate is picked up by astrocytic glutamate transpoter (GLT-1) where it undergoes one of the following fates: (1) gets converted by the glutamine synthetase (GS) enzyme to glutamine (Gln), (2) enters the TCA cycle as alpha ketoglutarate (alpha-KG) by the glutamate dehydrogenase enzyme, or (3) gets released in the perisynaptic space in exchange for cystine. Astrocytic Gln is shuttled to neurons where it gets converted to Glu by the phosphate-activated glutaminase (GA) enzyme. Neuronal Glu can also be generated through TCA cycle alpha-KG by the glutamate dehydrogenase enzyme. Neuronal Glu gets packaged into vesicles ready for release into the synapse during neuronal firing.

Most of the synaptic glutamate is removed into astrocytes by GLT-1 (EAAT2), and the astrocytic glutamate is converted *via* the glutamine synthetase (GS) enzyme into glutamine (Hertz, 2013), which is transported into pre-synaptic glutamatergic neurons and converted back to glutamate by the glutaminase (GA) enzyme. Glutamine is also transported into GABAergic neurons where it is converted into glutamate, which is biosynthesized to GABA by glutamic acid decarboxylase through TCA cycle (Bak et al., 2006; Hertz, 2013; Figure 1). This glutamate/glutamine/GABA cycle in glutamatergic or GABAergic systems has been found to be dysregulated in preclinical models (Rappeneau et al., 2016) and humans with neurological disorders (El-Ansary and Al-Ayadhi, 2014; Ford et al., 2017).

Dopaminergic neurons also express inotropic glutamate receptors (iGluRs), and blocking these receptors can lead to decrease in dopamine release (Desce et al., 1992; Fu et al., 2000). Conversely, glutamatergic neurons express dopamine receptors, which are stimulated by dopamine, to produce downstream signaling and intracellular postsynaptic effects (Yin and Lovinger, 2006). This indicates that the glutamatergic system has a variety of actions and functions, and that dysregulation of this system can affect other systems such as dopaminergic and GABAergic systems. In addition, glutamate/cystine antiporter (xCT) is another astrocytic glutamate transporter that is suggested to have neuroprotective effects by increasing the level of astrocytic glutathione (Seib et al., 2011).

## Role of Glutamate Transporters in Glutamate Homeostasis

Synaptic glutamate concentration is mainly regulated by astrocytic glutamate transporters in the mesocorticolimbic system. It was found that the GLT-1 in astrocytes plays a role in neuroprotection against neuro-excitotoxicity, and that neuronal GLT-1 is essential for glutamate uptake (Danbolt, 2001; Petr et al., 2015). This is in agreement with another study demonstrating that extracellular glutamate concentrations were decreased in synGLT-1 KO synaptosomes, indicating that glutamate homeostasis is regulated by GLT-1 (McNair et al., 2019). Moreover, it was reported that a hippocampal injury develops because of loss of neuronal GLT-1 (Rimmele et al., 2021). Importantly, GLT-1 is more highly localized in the forebrain than in the cerebellum (Holmseth et al., 2012). This was supported by studies that found a decrease in GLT-1 expression in the forebrain of preclinical models of neurological disorders (Ketheeswaranathan et al., 2011; Petr et al., 2013; Goodwani et al., 2015; Hakami et al., 2016). Downregulation of GLT-1 in the brain of ethanol exposed rats was associated with increase in synaptic glutamate level, which further indicates the role of GLT-1 in uptake of the majority of extracellular glutamate levels (Das et al., 2015). Moreover, studies suggested that upregulation of GLT-1 could be a potential therapeutic strategy to attenuate ethanol dependence and withdrawal and brain ischemia (Abulseoud et al., 2014; Das et al., 2015; Hu et al., 2015; Alasmari et al., 2016).

Additionally, glutamate is released from astrocytes through xCT and regulates glutamate neurotransmission by activating metabotropic glutamate receptor 2/3 (mGluR2/3) (Moran et al., 2005). The activity of xCT has been found to be correlated with the tone of glutamate on pre-synaptic mGluR2/3 (Preedy, 2016). This receptor controls the release of glutamate from pre-synaptic glutamate neurons through negative feedback cascade (Moussawi and Kalivas, 2010). Studies found that stimulating mGluR2/3 reduced nicotine-, ethanol-, and methamphetamine-seeking behaviors (Crawford et al., 2013; Justinova et al., 2016; Windisch and Czachowski, 2018). Alternatively, xCT transports cystine into astrocytes, is converted to cysteine, and leads to biosynthesis of glutathione (GSH) in astrocytes (Shih et al., 2003). Increase in extracellular glutamate level induces inhibition of cysteine uptake, reducing the biosynthesis of GSH, which leads to oxidative stress (Murphy et al., 1989). Therefore, astrocytic glutamate transporters, including GLT-1 and xCT, play a substantial role in regulating glutamate levels as well as the functional integrity of neurons.

## Glutamate Signaling Dysregulation in Animal Models of Psychiatric Disorders and Neurological Diseases

Accumulation of glutamate in the synaptic cleft due to excessive release or reduced uptake could be neurotoxic as a consequence of the hyperglutamatergic state, and this involves overstimulation of NMDA receptors causing calcium (Ca^2+^) influx and activation of apoptotic genes (Nishizawa, 2001; Guo et al., 2017; Sheets, 2017). Stimulation of Ca^2+^influx has been found to increase the progression of neurological diseases such as ischemic stroke and Alzheimer’s disease (Lopez et al., 2008; Tang et al., 2015). Studies have reported that the glutamate excitotoxicity observed in neurological disease models has been found to be associated with overstimulation of glutamate receptors in post-synaptic neurons such as NMDA receptors and AMPA receptors and their signaling pathways (Zhang et al., 2013).

The electrochemical gradient of Na^+^ and K^+^ is involved in the transport of glutamate through astrocytic glutamate transporters (Rose et al., 2009). In addition to Ca^2+^, glutamate can also affect other signaling pathways such as Na^+^ and K^+^ channels, which might be involved in the pathogenesis of neuropsychiatric disorders including schizophrenia, seizure, and acute ischemia (Lysko et al., 1994; Farber et al., 2002; Wang et al., 2004; Inyushin et al., 2010). Blocking Na^+^ channels may prevent the neurotoxicity induced by the NMDA blocker (Farber et al., 2002).

Additionally, the mammalian target of rapamycin (mTOR) is connected to the glutamatergic system through mGluR1/5 (Stoppel et al., 2017). Activating the mGluR1/5-mTOR signaling cascade by glutamate produced synaptic proteins (PSD-95 and GluR subunits of AMPAR) (Dayas et al., 2012). Studies have found that mTOR is involved in the psychiatric disorder-induced hyperglutamatergic state such as certain types of depression (Crino, 2016). mTOR is also stimulated by another signaling pathway, extracellular signal-regulated kinase (ERK) (Markworth and Cameron-Smith, 2011; Montana et al., 2014). Brain-derived neurotrophic factor (BDNF) stimulates tropomyosin receptor kinase B (TrkB) in post-synaptic neurons and is involved in activating the ERK-mTOR signaling pathway (Hua et al., 2016). These signaling systems of the glutamatergic system have been found to be altered in preclinical models of neurological disorders.

With regard to the NMDA- Ca^2+^ pathway, neuronal nitric oxide synthase (nNOS) has been found to be stimulated by intracellular Ca^2+^-calmodulin (Lee and Stull, 1998; Toda et al., 2009). This enzyme produces nitric oxide (NO) in post-synaptic neurons and is involved in stimulating soluble guanylate cyclase (sGC) and consequently activating signaling pathways such as cyclic guanylate and adenine monophosphate (cGMP or cAMP) (Liddie et al., 2013). Stimulation of both cGMP and cAMP can lead to increase in transcription of phosphorylated cyclic AMP response element-binding protein (pCREB) (Gallo and Iadecola, 2011). This post-synaptic NO signaling pathway has also been found in pre-synaptic neurons, since NO is transported into pre-synaptic neurons through the retrograde messenger system, and this can lead to further release of glutamate (Liddie et al., 2013). Additionally, NO is also transported into astrocytes, blocks the activity of the GS enzyme, and further increases glutamate concentrations (Kosenko et al., 2003). Thus, dysregulation of the NO system in the brain can cause glutamate accumulation and release, which may lead to glutamate neuroexcitotoxicity. It is important to note that nNOS activity was increased significantly in ischemic stroke modles, which may lead to neuroexcitotoxicity of glutamate in mesocorticolimbic areas (Eliasson et al., 1999).

## Evidence That Hyperglutamatergic State Causes Neuroexcitotoxicity

In the late 1960s and early 1970s, it was found that sustained exposure to glutamate and other excitatory amino acids may cause neuronal damage [for review refer to Choi (1988)]. The neurotoxicity observed in limbic seizure syndromes is ultrastructurally indistinguishable from the neuroexcitotoxicity induced by increased extracellular glutamate, and it can be blocked by NMDA receptor antagonists such as phencyclidine and ketamine (Olney, 1986). Glutamate neuroexcitotoxicity can be acute as in ischemic stroke or seizure or chronic as in neurodegenerative disorders, including amyotrophic lateral sclerosis, Alzheimer’s disease, and Huntington’s disease [for review refer to Doble (1999); Lewerenz and Maher (2015)]. Rothstein et al. developed a model of slow toxicity in cultured organotypic spinal cord slices. The model was based on selective inhibition of GLT-1, which continuously raised the concentration of glutamate in the culture media. This resulted in slow degeneration of motor neurons over several weeks. Motor neuron toxicity was selectively prevented by non-N-methyl-D-aspartate glutamate receptor antagonists and glutamate synthesis but not by N-methyl-D-aspartate receptor antagonists (Rothstein et al., 1993). Similar results were obtained in primary mouse cortical neurons, where the cell death resulting from short-term application of glutamate could be divided into NMDA-dependent and NMDA-independent phases. The NMDA receptor-independent component is associated with high extracellular glutamate and is inhibited by a variety of reagents that block the oxidative stress associated with glutamate neuroexcitotoxicity (Schubert and Piasecki, 2001). In addition, application of excitotoxins such as glutamate, N-methyl-D-aspartate (NMDA), and kainate (KA) to an organotypic slice culture of chick embryo spinal cord (Brunet et al., 2009), or blocking GLT-1 by threo hydroxyaspartate (THA) and L-trans-pyrrolidine-2, 4-dicarboxylate (PDC) in an organotypic rat lumbar spinal cord culture resulted in dose-dependent slow progression of neurodegeneration of spinal motoneurons (Matyja et al., 2005). It is important here to note that GLT-1 is expressed in presynaptic terminals of glutamatergic neurons as well as in astrocytes. Rimmele et al. (2021) provided evidence that the GLT-1 expressed in presynaptic neuronal terminals serves an important role in regulation of vulnerability to excitotoxicity. Stimulus-evoked field extracellular postsynaptic potentials recorded in the CA1 region of the hippocampus of conditional GLT-1 knockout (KO) mouse lines were normal in the astrocytic GLT-1 KO with elevated glutamate and were reduced in the neuronal GLT-1 KO but with normal glutamate accumulation.

### Acute Hyperglutamatergic States in Ischemic Stroke and Status Epilepticus

Patients with ischemic cerebral infarction showed significantly elevated plasma and CSF glutamate concentrations within 24 h of symptom onset (Castillo et al., 1996). GLT-1 mRNA expression was decreased significantly in the hippocampus, cortex, and striatum in a mouse model of focal ischemic stroke induced by middle cerebral artery occlusion (Ketheeswaranathan et al., 2011). Furthermore, animal models of status epilepticus induced by LiCl-pilocarpine administration showed NMDA-mediated glutamatergic neuroexcitotoxicity in the CA1 hippocampal subfield, habenula, thalamus, and amygdala that was successfully blocked by the NMDA receptor antagonist ketamine (Loss et al., 2012).

### Chronic Hyperglutamatergic States in Certain Neurodegenerative Disorders

#### Amyotrophic Lateral Sclerosis

Infusion of NMDA receptor agonists directly onto the lumbar spinal cord of 21-day old rats following posterior laminectomy caused changes in dorsal horn neurons, while infusion of a non-NMDA agonist, kainic acid, affected motor neurons, and the observed changes in motor axons resemble the changes described in the spinal cord of patients with ALS (Ikonomidou et al., 1996). In addition, a transgenic superoxide dismutase 1 (SOD1) mutant rat model of ALS showed focal loss of GLT-1 in the ventral horn of the spinal cord prior to the onset of motor neuron/axon degeneration (Howland et al., 2002), and there is evidence for decreased brain tissue concentration of glutamate and increased levels in the CSF of patients with ALS [for review refer to Heath and Shaw (2002)]. Ceftriaxone upregulated GLT-1, attenuated ALS manifestations in a mouse model (Rothstein et al., 2005), increased system x(c)(-) and glutathione levels in rat cortical and spinal astrocytes and fibroblasts and hippocampal cell lines, and induced xCT mRNA expression in stem cell-derived human motor neurons (Lewerenz et al., 2009).

#### Alzheimer’s Disease

CSF glutamate levels are significantly increased in patients with Alzheimer’s disease compared to healthy controls in some (Pomara et al., 1992; Csernansky et al., 1996) but not all studies (Martinez et al., 1993; Kuiper et al., 2000), and a 3xTg-AD mouse model shows age-dependent progressive reduction in hippocampal GLT-1 levels (Zumkehr et al., 2015). Furthermore, Gray and Patel (1995) showed that adding glutamate to a neuronal culture for 15 min resulted in dose-dependent neuronal degeneration, and that the presence of astrocytes decreased the excitotoxicity of glutamate in neurons because of uptake of glutamate by astrocytes. The latter study showed that incorporation of an NMDA receptor-mediated Ca^2+^ ion channel blocker, MK-801, together with glutamate completely inhibited the degeneration of cortical neurons (Gray and Patel, 1995). Glutamate excitotoxicity is attributed, at least in part, to destabilization of neuronal calcium regulation by beta-amyloid proteins (Mattson et al., 1992; Kim et al., 2000).

Brain beta-amyloid deposition in Alzheimer’s disease follows a distinct spatial progression starting from the basal neocortex, to the hippocampus, and then to the rest of the cortex (Raskin et al., 2015). In addition, beta-amyloid interrupts effective glutamate uptake by GLT-1 (Conway, 2020), and microinjection of beta-amyloid fibrils into the hippocampal CA1 area of rats is associated with reduction in GLT-1 (Wu et al., 2020). A recent study conducted chemical exchange saturation transfer imaging of glutamate (gluCEST) to characterize the distribution of the gluCEST contrast in the whole brain and in large-scale networks of mouse lemur primates as a model of Alzheimer’s disease (Garin et al., 2022). This showed the detection of high gluCEST contrast in the nucleus accumbens, septum, basal forebrain, and cortical areas 24 and 25. Furthermore, the study was able to identify an age-related decrease in the gluCEST contrast in the nucleus accumbens, septum, basal forebrain, globus pallidus, hypophysis, cortical areas 24, 21, and 6, and in olfactory bulbs. An age-related gluCEST contrast decrease was also detected in specific neuronal networks such as fronto-temporal and evaluative limbic networks (Garin et al., 2022).

#### Huntington’s Disease

Systemic administration of monosodium glutamate to immature mice causes degeneration of certain neurons of retina and brain (Coyle and Schwarcz, 1976) and GLT1mRNA decreased in postmortem neostriatum of HD in correlation to disease severity and loss of neurons with NMDA receptors in the neostriatum of HD (Arzberger et al., 1997). However, GLT-1 expression does not necessarily translate to accelerated glutamate uptake. Wilkie et al. (2020) showed an activity-dependent increase in extracellular glutamate accumulation in the HD hippocampus that was not associated with reduced GLT-1 expression and was not reduced by ceftriaxone administration. Furthermore, ceftriaxone administration to wild-type mice increased GLT-1 expression in multiple brain regions but did not prevent the activity-dependent slowing of glutamate clearance in the striatum (Wilkie et al., 2021). Along the same lines, changes in GLT-1 expression or function per se are unlikely to potentiate or ameliorate the progression of HD. Petr et al. (2013) generated a heterozygous null allele of GLT-1 that also carries the R6/2 transgene (double mutation) as a model for HD. The mice showed no exacerbation of clinical manifestations (weight loss, accelerating rotarod, climbing, and paw-clasping), and there was no change in striatal glutamate uptake compared to the wild-type mice. The further skepticism about impairment in glutamate uptake as a core pathology of HD came from studies showing that real-time measures of glutamate clearance in the striatum of an R6/2 model of HD are normal or even accelerated despite impaired synaptosomal glutamate uptake (Parsons et al., 2016).

#### Argument Against the Hypothesis That Glutamate Transporter 1 Dysfunction Causes Hyperglutamatergic State

Glutamate transporter 1 expression does not necessarily translate to accelerated glutamate uptake. Activity-dependent increase in extracellular glutamate accumulation in the HD hippocampus was not associated with reduced GLT-1 expression and was not reduced by CEF administration (Wilkie et al., 2020). Furthermore, CEF administration to wild-type mice increased GLT-1 expression in multiple brain regions but did not prevent activity-dependent slowing of glutamate clearance in the striatum (Wilkie et al., 2021).

#### Role of Extrasynaptic Glutamate in Excitotoxicity

N-methyl D-aspartate receptors (NMDARs) are located in synaptic and extrasynaptic sites. Calcium influx through synaptic NMDARs induced synaptic plasticity by induction of cAMP response element-binding protein (CREB) activity and brain-derived neurotrophic factor (BDNF) gene expression, while calcium influx through extrasynaptic NMDARs causes neuroexcitotoxicity (Hardingham et al., 2002; Vanhoutte and Bading, 2003; Papouin et al., 2012; Papouin and Oliet, 2014). Extracellular glutamate is regulated by the glutamate/cystine antiporter system x(c), which transports glutamate in exchange for cystine. Intracellular cystine is transformed into the antioxidant glutathione. Reduced activity of system x(c) can lead to neurotoxicity because of reduced intracellular glutathione (Albrecht et al., 2010). Inhibition of glutathione either directly by exposing a hippocampal cell culture to L-trans-pyrrolidine-2,4-dicarboxylate or indirectly by inhibiting GLT-1 by threo-beta-benzyloxyaspartate increased neuronal death more than by exposure to glutamate alone (Himi et al., 2003). Interestingly, young mice lacking the specific xCT subunit of system x(c)-(xCT(−/−)) had significantly lower extracellular hippocampal glutamate concentrations associated with spatial working memory deficit, but there was no reduction in hippocampal glutathione content compared to wild-type littermates (De Bundel et al., 2011).

## Regulation of Hyperglutamatergic State With Ceftriaxone

Based on the argument that hyperglutamatergic state contributes to the development of several neurological and psychiatric diseases, decrease in GLT-1 and xCT expression can lead to hyperglutamatergic state, and upregulating these transporters by the β-lactam antibiotic CEF, known to upregulate GLT-1 and xCT, could be a potential pharmacological approach to attenuate glutamate dysregulation (Rothstein et al., 2005; Sari et al., 2011; Das et al., 2015). We discussed here the effectiveness of CEF in attenuating the symptom severity and clinical manifestations of neurological disorders and psychiatric diseases, which are caused by hyperglutamatergic state.

### Efficacy of Ceftriaxone in Preclinical Models of Neurological Disorders

#### Effect of Ceftriaxone in Ischemic Stroke Animal Model

Ceftriaxone, pre- (for 5 days) or post-treatment (for 5 days), induced neuroprotection in part by upregulation of GLT-1 expression in brain ischemic male Wistar rats (Hu et al., 2015). This effect was not observed in animals injected with dihydrokainic acid (GLT-1 blocker) or antisense oligodeoxynucleotides, which indicates that GLT-1 plays a major role in neuronal damage associated with ischemic stroke. Furthermore, one study reported loss in glutamate transport activity and GLT-1 expression in CA1 hippocampal neurons within few hours of induction of transient forebrain ischemia, and CEF protected the CA1 neurons (Ouyang et al., 2007). Repeated administration of CEF (200 mg/kg/day) for 5 days prior to ischemia reduced the infarction volume in Sprague-Dawley male rats (Chu et al., 2007). However, this study found that post-treatment with CEF did not affect the level of infarction volume. Furthermore, studies reported that focal cerebral ischemia increased glutamate levels and reduced the expression of GLT-1 and xCT in the frontal cortex and hippocampus, and that CEF (200 mg/kg/day) for 5 days pre-cerebral ischemia attenuated these effects (Krzyżanowska et al., 2017). Together, these findings indicate that pre-treatment with CEF at an adequate dose (200 mg/kg/d) for 2-5 days prior to induction of hyperglutamatergic state shows better ischemic recovery than post-treatment. This observation highlights that CEF does not reverse an already established neuronal damage induced by prolonged hyperglutamatergic excitotoxicity state.

Mechanistically, CEF injection for 5 days pre-induction of cerebral ischemia increased the mRNA and protein expression of GLT-1 as well as glutamate uptake and GS enzyme activity (Verma et al., 2010). This effect was associated with decrease in infarction size and neuronal loss. This study suggests that modulation of glutamate homeostasis by CEF may induce neuroprotection in ischemic models. Moreover, a single injection of CEF (200 mg/kg/day) was able to reduce the mortality rate, infract size, and neuronal death in a stroke rat model (Thöne-Reineke et al., 2008). This effect was associated with increase in BDNF and TrkB expression in the brain, indicating the involvement of the BDNF-TrkB pathway in the effectiveness of CEF in ischemic stroke. However, there is evidence showing that reversal of glutamate transporters, including GLT-1, is important in causing excitotoxicity in ischemia. It has been shown in rat hippocampal slices exposed to oxygen and glucose deprivation as a model of acute ischemia that glutamate release is blocked by pretreatment with two different competitive substrate analogues of the Na(^+^)-dependent glutamate transporters (Roettger and Lipton, 1996). In another model of acute ischemia induced by bilateral carotid artery occlusion and controlled hypotension in rats, ischemia-associated increase in striatal glutamate was attenuated by acute infusion of GLT-1 blocker dihydrokainate (Seki et al., 1999). We suggest that the effect is transitory on modulating the release of glutamate by acute exposure to the GLT-1 blocker in this ischemic model, and that chronic exposure to CEF is the key for normalizing GLT-1 expression to regulate glutamate homeostasis in the ischemic state.

#### Effect of Ceftriaxone on Animal Models of Amyotrophic Lateral Sclerosis (ALS) and Multiple Sclerosis

Rothstein et al. reported a significant reduction in the maximal velocity of transport for high-affinity glutamate uptake in synaptosomes collected from the spinal cord, motor cortex, and somatosensory cortex of patients with ALS (Rothstein et al., 1992). Furthermore, a study from the same laboratory reported that β-lactam antibiotics upregulated GLT-1 protein expression in a rodent lumbar spinal cord culture and activated the human GLT-1 promoter in human fetal astrocytes transfected with the GLT-1 promoter/luciferase reporter (Rothstein et al., 2005). In addition, all β-lactam antibiotics activated the GLT-1 promoter and protein expression *in vivo*, and CEF pretreatment was protective in two different models, delayed disease onset and loss of muscle strength, and increased survival in ALS mice (Rothstein et al., 2005). This groundbreaking study prompted the initiation of a large-scale, multi-center clinical study testing the efficacy of CEF treatment in slowing the functional decline and improving the overall survival of patients with ALS (Cudkowicz et al., 2014). We will discuss this study in more details in section “Clinical Studies Testing the Efficacy of Ceftriaxone”.

Regarding multiple sclerosis, one study reported that CEF(200 mg/kg/day) administered to an MOG peptide immunization mouse model of MS either from the day of immunization (permanent) or from the onset of motor manifestations (therapeutic) (Melzer et al., 2008) showed better motor outcomes than vehicle treatment. However, early CEF administration (permanent) improved motor outcome significantly better than late administration (therapeutic treatment). It is important to note that CEF’s mechanism of action could be attributed to attenuation of inflammation-induced excitotoxicity associated with MS by slowing down the production of pro-inflammatory mediators (IL-17 and interferon gamma (IFNγ)) and proliferation of T cells (Melzer et al., 2008), as we discuss later in section “Attenuation of Pro-inflammatory Cytokines”.

#### Effect of Ceftriaxone in Animal Models of Huntington’s Disease

Miller et al. (2008) demonstrated that treatment with CEF (200 mg/kg/day) for 5 days reduced HD-associated behaviors in mice. This effect was associated with increase in GLT-1 expression and decrease in extracellular glutamate concentration as compared to a control HD mouse model. Moreover, another study demonstrated a decrease in GLT-1 expression in the striatum and cerebral cortex if an HD mouse model compared to wild-type mice at the age of 13 weeks but not 9 weeks, and CEF treatment (200 mg/kg/day) for 5 days restored GLT-1 expression in both brain regions (Sari et al., 2010). Taken together, these data suggest that HD is associated with decrease in GLT-1 expression and increase in extracellular glutamate concentration in affected brain regions including the striatum.

#### Effect of Ceftriaxone in Animal Models of Seizure

Studies have shown that CEF attenuated seizure by upregulating GLT-1 expression. For instance, a significant reduction in GLT-1 protein expression was found in the ipsilesional cortex 7 days after traumatic brain injury in rats (Goodrich et al., 2013). This study reported that CEF (200 mg/kg/day) for 7 days following the injury increased GLT-1 expression and reduced the duration of traumatic seizure. Additionally, incubation of CEF at 3, 5, 10, and 25 mM reduced cocaine-induced planarian seizure-like activity in *Dugesia dorotocephala* (Rawls et al., 2010b). Furthermore, CEF reduced the duration of seizure through non-glutamatergic pathways. Post-treatment with CEF (200 mg/kg) twice daily for 3 days increased onset latency and decreased the duration of pentylenetetrazole (PTZ)-induced seizure in rats (Hussein et al., 2016). This effect was associated with decrease in connexin 43 expression, increase in GSH content, and increase in catalase activity in the brain. In addition to post-treatment, pre-treatment with CEF (200 mg/kg/day) for 6 days has been found to attenuate generalized clonic-tonic convulsions induced by PTZ in mice (Jelenkovic et al., 2008).

#### Effect of Ceftriaxone on Cognitive Impairment

Ceftriaxone treatment significantly alleviated the cognitive deficits measured by Morris water maze test and upregulated GLT-1 protein expression in the hippocampus of APP/PS1 mice (Fan et al., 2018). Particularly, the activity of glutamine synthetase (GS) and the protein expression of system N glutamine transporter 1 (SN1), which are the key factors involved in the glutamate-glutamine cycle, were significantly upregulated, and inhibition of GLT-1 uptake activity by dihydrokainic acid, an inhibitor of GLT-1, blocked CEF-induced improvement in cognitive deficits, GS activity, and SN1 expression (Fan et al., 2018). Furthermore, daily treatment with CEF (200 mg/kg) for 2 months has been reported to attenuate the accumulation of pathological tau, and this effect was associated with increase in GLT-1 expression in the hippocampus in a 3xTg-Alzheimer’s disease mouse model (Zumkehr et al., 2015). Another study suggested that CEF could improve the cognitive impairments of APP/PS1 mice in the early stage of Alzheimer’s disease by upregulating GLT-1 and Nglutamine transporter 1 (SN1) expression and increasing the activity of the GS enzyme (Fan et al., 2018). Moreover, studies showed that CEF attenuated the impairments of cognitive function in a Parkinson’s disease (PD) rat model (Ho et al., 2014; Hsu et al., 2015). For example, CEF (200 mg/kg/day) for 14 days improved the cognitive function and memory of a PD rat model (Ho et al., 2014). This effect was associated with improvements in microglial activation or degeneration of dopaminergic neurons in specific brain areas. Furthermore, it has been demonstrated that CEF attenuated cognitive dysfunction, hyperactivity of glutamatergic neurons, and hippocampal CA1 neuronal loss in a PD rat model. This study found a significant increase in GLT-1 expression in the striatum and hippocampus following treatment with CEF (Hsu et al., 2015). Furthermore, it has been demonstrated that administration of CEF at a lower dose (100 mg/kg, IP) for 27 days improved cognitive function and increased neurogenesis in the brains of a rat model of dementia with Lewy bodies (DLBs) (Ho et al., 2019). Taken together, it seems that CEF treatment associated with cognitive function improvement in animal models of different cognitive impairments is reported after extended treatment (14-60 days) and other mechanisms of action might be involved besides GLT-1 upregulation such as increase in the activity of the GS enzyme, increase in neurogenesis, and decrease in microglial activation.

#### Efficacy of Ceftriaxone in Animal Models of Pain Disorders

##### Visceral Pain, Hyperalgesia, and Allodynia

Dysregulation of GLT-1 has been observed in animal models of visceral pain (Lin et al., 2009). Ceftriaxone treatment (200 mg/kg/day) for 1 week decreased the response of visceral nociceptive in mice (Lin et al., 2009; Ackerman et al., 2015). In addition to attenuation of visceral pain, it has been found that CEF upregulated GLT-1 expression in the spinal cord (Ackerman et al., 2016). Thus, astrocytic glutamate transporters could be pharmacologically targeted to attenuate visceral pain. Moreover, tactile allodynia and visceral hyperalgesia have been found in female Wistar-Kyoto rats 3, 5, 8, and 10 days after induction of stress, and this effect was correlated with reduced GLT-1 expression in the spinal cord (Ackerman et al., 2015). Downregulation of GLT-1 by dihydrokainate also induced a similar effect. This effect was attenuated by upregulation of GLT-1 in the spinal cord following treatment with CEF (200 mg/kg IP) for 5 days (Ackerman et al., 2015). Furthermore, a study evaluated the effects of CEF (200 mg/kg IP) treatment for 7 days on nicotine (1 or 2.5 mg/kg sc) antinociception and its tolerance in rats by tail flick assay. In that study, CEF-treated rats displayed an enhanced antinociceptive response to nicotine and did not develop tolerance to nicotine’s analgesic effects (Schroeder et al., 2011).

##### Neuropathic Pain

In addition to visceral pain, hyperalgesia, and allodynia, CEF treatment was reported to attenuate hyperalgesia in neuropathic pain models by upregulation of glutamate transporters (Hu et al., 2015). Chronic constrictive injury (CCI) induced a neuropathy characterized by mechanical allodynia and hyperalgesia (Hu et al., 2015). This study also found that CCI downregulated GLT-1 expression in the spinal dorsal horn. CEF treatment (200 mg/kg/day IP) for 7 days showed the ability to reduce mechanical allodynia and thermal hyperalgesia induced by CCI by upregulating GLT-1 (Hu et al., 2015). Furthermore, treatment with CEF (100 mg/kg IP) alone or in combination with pioglitazone prevented neuropathic pain and biochemical, mitochondrial, and cellular alterations in the spinal cord of ligated spinal nerves in rats (Pottabathini et al., 2016). In addition, CEF treatment (200 mg/kg/day) for 7 days caused a significant attenuation of cold-induced tactile allodynia in oxaliplatin-treated mice without inducing adverse motor effects on the mice (Salat et al., 2019). Therefore, GLT-1 could be pharmacologically targeted to attenuate neuropathic pain.

##### Morphine Allodynia and Analgesia Tolerance

A study reported that the analgesic effect of morphine is mediated in part by the modulatory action of GLT-1 (Rawls et al., 2010c). This study found that CEF treatments (50, 100, and 200 mg/kg) 15 min before each morphine injection twice daily for 7 days attenuated morphine-induced tolerance, and this effect was abolished following the administration of a GLT-1 inhibitor, dihydrokainate. These observations confirm that GLT-1 signaling pathways could be targeted to attenuate tolerance induced by morphine. Furthermore, a study from the same group provided additional findings that showed pre-treatment with CEF (200 mg/kg/day) for 7 days reduced hyperthermia induced by morphine exposure (Rawls et al., 2007).

### Efficacy of Ceftriaxone in Preclinical Models of Psychiatric Disorders

#### Depression

Recent studies indicate that GLT-1 is involved in the development of depression-like symptoms in animal models (John et al., 2015; Rappeneau et al., 2016; Ding et al., 2017). For instance, blockage of GLT-1 in the central amygdala was found to be associated with depressive manifestations in rats (John et al., 2015). A marked disruption in the glutamate-glutamine system has been observed in male and female rat models of depression (Rappeneau et al., 2016). CEF-treated animals (200 mg/kg/d) for 14-18 days also showed a marked decrement in immobility in the forced swim and tail suspension tests. Even though not statistically significant, a similar trend was noted in novelty-suppressed feeding (Mineur et al., 2007). Moreover, CEF (200 mg/kg/d) for 5 days before transient occlusion of common carotid arteries to induce transient brain ischemia in pregnant rats attenuated the development of postpartum depression by preventing the loss of GLT-1 expression in the mPFC (Guan et al., 2016). Similarly, CEF (200 mg/kg/d) for 5 days significantly increased GLT-1 expression and normalized the firing of lateral habenula (LHb) neurons and alleviated a depression-like behavior in ethanol-withdrawn rats (Kang et al., 2018). These studies lend more evidence to the concept that repeated CEF administration before initiating a stressor or brain change that is associated with depressive-like behaviors is associated with upregulation of GLT-1 and attenuation of behavioral manifestations.

#### Substance Use Disorders

##### Cocaine

Changes in astrocytic GLT-1 and glutamatergic receptor expression have been observed in animals exposed to cocaine (Hammad et al., 2017a,b). Thus, repeated cocaine administration reduced the expression of GLT-1 and xCT in the NAc (Hammad et al., 2017b). Additionally, reinstatement of cocaine was associated with reduction in GLT-1 and xCT expression in the NAc core and shell (Hammad et al., 2017a).

The effect of CEF pretreatment in cocaine intake remains controversial. While CEF 200 mg/kg/d for 3 days prior to first cocaine exposure blocked the ability of mice to acquire cocaine in a conditioned place preference (CPP) paradigm (Ward et al., 2011), another study reported that CEF 200 mg/kg/d for 5 days prior to first cocaine exposure did not affect the acquisition of cocaine self-administration (Sondheimer and Knackstedt, 2011). In contrast, several studies document the efficacy of CEF in preventing cocaine relapse. We have shown that the presentation of cues (light and tone) previously associated with cocaine self-administration reinstated lever pressing in rats treated with vehicle, whereas CEF (100 or 200 but not 50 mg/kg/d × 5 days during extension) blocked this response (Sari et al., 2009). Similar results were reported by Kalivas’ laboratory where CEF 200 mg/kg/day × 7 days during extinction prevented both cue- and cocaine-induced reinstatement of drug-seeking (Knackstedt et al., 2010), and that the reinstatement-blocking effect lasted for weeks after the CEF treatment ceased (Sondheimer and Knackstedt, 2011). In addition, CEF (200mg/kg/day) attenuated cue-induced cocaine-seeking behavior only in rats exposed to long (45 days) withdrawal periods, with greater effect on the extended-access condition (6 h/day) compared to those with short withdrawal (2 days) and limited access (2 h/day) (Fischer et al., 2013). In addition, CEF may attenuate cue extinction only when such extinction occurs in the same context as cocaine self-administration. However, when cue extinction is conducted outside the drug-associated context, it does not reduce the risk of relapse alone (Bechard and Knackstedt, 2019).

The efficacy of CEF in blocking cocaine reinstatement is attributed to upregulation of GLT-1 and xCT levels (Sari et al., 2009; Knackstedt et al., 2010) in the PFC and NAc. A recent study suggested that upregulating both GLT-1 and xCT is required. Intra-NAcxCT knockdown prevented the effects of CEF on attenuating reinstatement and upregulating GLT-1 and resulted in increased surface expression of AMPA receptors; intra-NAc GLT-1 knockdown prevented the effects of CEF on attenuating reinstatement and upregulating xCT expression without affecting AMPA receptor expression (LaCrosse et al., 2017). In addition, intra-NAc administration of the mGlu2/3 receptor antagonist during cue- and cocaine-primed reinstatement tests prevented the effects of CEF on attenuating reinstatement, suggesting that CEF also works by activating mGlu 2/3 receptors (Logan et al., 2020) and preventing NAc core glutamate efflux likely by reducing activity in prelimbic NAc core-projecting neurons (Bechard et al., 2021). We suggest here that there is a different pattern where initiating CEF prior to cocaine administration does not seem to block the acquisition of cocaine self-administration. However, it prevented cocaine preference in the CPP paradigm. Alternatively, CEF administration after animals received cocaine prevented relapse in some but not all conditions. This variability in response could be attributed to non-glutamatergic mechanisms of cocaine such as its effect on inhibiting acetylcholine receptor-controlled ion flux in sympathetic neuronal cell lines (Karpen et al., 1982).

##### Opiate

Astrocytic GLT-1 has been found to be modulated by morphine or morphine-like compounds in mesocorticolimbic brain regions (Ozawa et al., 2001; Fujio et al., 2005; Tai et al., 2007; Alshehri et al., 2018). Alteration of astrocytic GLT-1 protein expression has been observed with opioid dependence, withdrawal, and reinstatement (Ozawa et al., 2001; Xu et al., 2003; Alshehri et al., 2018). Therefore, astrocytic GLT-1 activity might be sensitive to morphine or morphine-like compounds. CEF at different doses ranging from 50 to 200 mg/kg/d administered for 4-7 days before morphine administration reduced the efficacy of morphine in causing hyperthermia in rats (Rawls et al., 2007) and decreased the development of tolerance to the antinociceptive effect of morphine (Habibi-Asl et al., 2014). Moreover, CEF inhibited naloxone-precipitated withdrawal manifestations (Rawls et al., 2010a; Habibi-Asl et al., 2014), reduced hydrocodone-induced reinstatement (Alshehri et al., 2018), and prevented cue-induced heroin-seeking (Shen et al., 2014). In addition to normalizing GLT-1, CEF restored glutamate uptake and prevented synaptic glutamate spillover in the NAc (Shen et al., 2014) and normalized xCT expression in NAc and the hippocampus (Alshehri et al., 2018). These studies suggest that CEF administration before exposure to opiates attenuated certain manifestations of opiate use such as hyperthermia and tolerance to the antinociceptive effect, and CEF administration after exposure to opiate reduced naloxone-precipitated withdrawal and prevented drug- and cue-triggered relapse in reinstatement animal models.

##### Ethanol

Ethanol self-administration has been found to increase extracellular glutamate concentration in the NAc (Gass et al., 2011). Studies from Sari’s laboratory showed that chronic consumption of ethanol (5 weeks) reduced the expression of GLT-1 in central reward brain regions including the NAc, amygdala, and hippocampus (Aal-Aaboda et al., 2015; Goodwani et al., 2015). Several studies, including those from Sari’s laboratory, have investigated the effects of ethanol exposure on extracellular glutamate concentration in the NAc (Melendez et al., 2005; Ding et al., 2013; Das et al., 2015). These studies reported that ethanol exposure increased extracellular glutamate concentration in the NAc. Interestingly, chronic intermittent inhalation of ethanol vapor has been reported to increase extracellular glutamate concentration in the NAc in mice (Griffin et al., 2015). However, continuous ethanol exposure decreased glutamate uptake in the NAc (Melendez et al., 2005; Das et al., 2015). This effect was associated with significant reduction of GLT-1 and xCT expression in the NAc in rats (Alhaddad et al., 2014a,b; Hakami et al., 2016). It is important to note that ethanol exposure induced the downregulation of GLT-1 expression in the NAc, amygdala, and hippocampus but not in the PFC [for review refer to Alasmari et al. (2018)] (Table 1).

**TABLE 1:** Effects of ethanol, MS-153, and β-lactam antibiotics on GLT-1 and NF*k*B expression in the nucleus accumbens (NAc), prefrontal cortex (PFC), amygdala (AMG), hippocampus (HIPP), and striatum (STR).

	Ethanol		MS-153		β-lactam antibiotics	
	GLT-1	NF*k*B	GLT-1	NF*k*B	GLT-1	NF*k*B
NAc	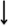 Alhaddad et al., 2014b; Goodwani et al., 2015	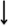 Alhaddad et al., 2014b	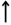 Alhaddad et al., 2014b	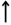 Alhaddad et al., 2014b	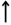 Rao et al., 2015a	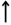 Rao et al., 2015b
PFC	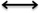 Alhaddad et al., 2014b	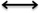 Alhaddad et al., 2014b	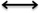 Alhaddad et al., 2014b	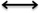 Alhaddad et al., 2014b	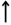 Rao et al., 2015a	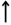 Rao et al., 2015b
AMG	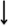 Aal-Aaboda et al., 2015	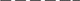	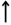 Aal-Aaboda et al., 2015	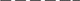	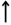 Rao and Sari, 2014a,b	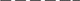
HIPP	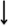 Aal-Aaboda et al., 2015; Alshehri et al., 2017	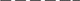	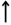 Aal-Aaboda et al., 2015	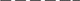	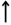 Alshehri et al., 2017	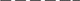
STR	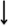 Alshehri et al., 2017	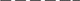	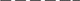	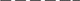	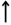 Alshehri et al., 2017	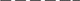

CEF (100-200 mg/kg) for 5-7 days after established ethanol intake reduced daily the ethanol consumption of adult and adolescent alcohol P rats (Sari et al., 2011, 2013a,b; Rao and Sari, 2014a; Das et al., 2015; Rao et al., 2015b) and Sprague-Dawley rats (Stennett et al., 2017). In addition, CEF treatments reduced the ethanol intake of type 1 equilibrative nucleoside transporter (ENT1) knockout mice (Lee et al., 2013), but not male C57BL/6 J mice (Griffin et al., 2021), and reduced or abolished all manifestations of ethanol withdrawal in both alcohol P rats and Wistar rats (Abulseoud et al., 2014) when administered at 100 mg/kg twice daily for 2 days at the onset of cessation of high-dose ethanol intake. Similarly, CEF resulted in recovery of exploratory behavioral patterns in a zebrafish ethanol withdrawal model (Agostini et al., 2020). In addition, CEF attenuated cue-primed reinstatement of ethanol-seeking in male Sprague-Dawley rats (Weiland et al., 2015), attenuated relapse-like ethanol-drinking behavior in alcohol P rats (Qrunfleh et al., 2013; Alhaddad et al., 2014a; Rao and Sari, 2014b), and prevented withdrawal-induced escalation of ethanol intake (Abulseoud et al., 2014). Mechanistically, CEF increased GLT-1 in most (Sari et al., 2013b; Abulseoud et al., 2014; Alhaddad et al., 2014a; Das et al., 2015; Rao et al., 2015b) but not all studies (Stennett et al., 2017; Griffin et al., 2021). Nuclear factor kappa-B (NF*k*B) has been found as one of the main signaling pathways involved in modulation of GLT-1 expression (Rao et al., 2015b). Chronic ethanol drinking reduced NF*k*B expression, and this effect was associated with reduction in GLT-1 expression (Alhaddad et al., 2014b). CEF enhanced the phosphorylation of Akt and nuclear translocation of nuclear NFκB in the NAc and PFC and normalized ethanol-induced increase in BDNF in NAc shell (Alhaddad et al., 2020). Furthermore, CEF also increased xCT (Alhaddad et al., 2014a; Rao and Sari, 2014a; Rao et al., 2015b; Stennett et al., 2017), ENT1, and aquaporin 4 (AQP4) expression in the striatum (Lee et al., 2013), PCF, and NAc core (Qrunfleh et al., 2013; Sari et al., 2013a) and the activity of the NAc GS enzyme but not in the PFC (Das et al., 2015). In addition, CEF normalized withdrawal-induced increase in reactive species (Agostini et al., 2020) as well as the hyperactivity of lateral habenula neurons in slices from withdrawn rats (Kang et al., 2018). Together, there is a rich body of literature showing that repeated CEF administration for 5-7 days several weeks after initiating ethanol drinking decreased ethanol drinking and prevented a relapse-like behavior. In our withdrawal study (Abulseoud et al., 2014), CEF was administered for only 2 days at the onset of cessation of high-dose ethanol intake, and this short treatment interval was enough to abolish ethanol withdrawal manifestations in two different strains of rats and reduce post-withdrawal ethanol consumption.

##### Amphetamine and Methamphetamine

Studies from Abulseoud and Sari’s laboratories reported that exposure to methamphetamine (METH) reduced the expression of GLT-1 in mesocorticolimbic brain regions (Abulseoud et al., 2012; Althobaiti et al., 2016, 2018). CEF treatment (200 mg/kg/day) for 7 days attenuated METH preference in a CPP paradigm at least in part by upregulation of GLT-1 expression (Abulseoud et al., 2012). Recently, Sari’s laboratory showed that upregulation of astrocytic glutamate transporters was found to reduce METH reinstatement in alcohol P rats (Althobaiti et al., 2018). CEF (200 mg/kg/day) was also found to restore the reduction of GLT-1 and xCT expression in the NAc caused by repeated exposure to high-dose METH of Wistar rats (Althobaiti et al., 2016). Furthermore, CEF (200 mg/kg/day) administered after daily extinction sessions reduced amphetamine-induced drug-seeking under enriched conditions and standard conditions but not in rats kept under isolated conditions (Garcia et al., 2019). Moreover, CEF attenuated amphetamine-induced hyperactivity and behavioral sensitization (Rasmussen et al., 2011) as well as the development of physical dependence and abstinence-induced withdrawal from amphetamine and METH using a planarian model (Rawls et al., 2008). In addition to its effect on reducing tissue glutamate and glutamine content, METH induces a significant depletion of dopamine and 5-HT in the NAc and PFC. Importantly, CEF treatment restores dopamine tissue content in the NAc (Althobaiti et al., 2018). Together, these studies demonstrate that CEF pretreatment for several days reduced preference and relapse to METH and attenuated amphetamine-induced hyperactivity and behavioral sensitization but not under isolation conditions and prevented withdrawal manifestations in a planarian model.

##### Cannabis

A body of evidence indicates that the endocannabinoid system interacts with the glutamatergic system in animals that have developed cannabis dependence. This is supported by findings showing that endocannabinoid controls the function of NMDA receptors (Sánchez-Blázquez et al., 2014; Rodríguez-Muñoz et al., 2016). In addition, exposure to a cannabinoid receptor 1 (CB1 receptor) agonist (CP 55,940) was associated with reduction in GLT-1 expression in the mesocorticolimbic system (Hakami et al., 2018). Ampicillin, a β lactam antibiotic (200 mg/kg/day), treatment attenuated reinstatement to CP 55,940 by upregulation of GLT-1 (Hakami et al., 2018). Furthermore, it has been found that CEF (100-200 mg/kg) significantly ameliorated the development of tolerance to the analgesic and hypothermic effects of a CB1 receptor agonist (WIN 55,212-2) without affecting its cataleptic action (Gunduz et al., 2011). We suggest here that with only two studies on CB1 agonists, not on THC itself, it is difficult to know whether CEF impacts the complex array of cannabis use-associated behaviors.

##### Nicotine

Chronic exposure to nicotine has been found to alter the expression of glutamatergic receptors and transporters as well as glutamate release (Wang et al., 2007; Knackstedt et al., 2009; Alasmari et al., 2017). Furthermore, chronic nicotine self-administration has been found to upregulate iGluRs in mesocorticolimbic brain regions (Wang et al., 2007; Kenny et al., 2009). Moreover, exposure to nicotine stimulated the release of glutamate in the PFC, and this effect was reduced by a nAChR antagonist, which indicates the modulatory role of nAChRs in nicotine-increased glutamate release (Konradsson-Geuken et al., 2009). Downregulation of GLT-1 expression in the striatum and xCT in the striatum and hippocampus has been found in mice exposed to e-cigarette vapors containing nicotine for 6 months (Alasmari et al., 2017). This effect was further supported by a study that showed that nicotine self-administration for 21 days reduced GLT-1 and xCT expression in the NAc (Knackstedt et al., 2009).

Importantly, CEF treatment (200 mg/kg/day) for 5 days decreased nicotine-seeking behavior, and this effect was associated with upregulation of GLT-1 expression in the NAc and PFC (Sari et al., 2016). CEF treatment (200 mg/kg/day) twice daily for 4 days reduced reinstatement to nicotine in mice using the CPP paradigm (Alajaji et al., 2013). We have shown that short-term nicotine abstinence is associated with reduced glutamate concentration in the dACC in humans (Abulseoud et al., 2020), and that CEF decreased nicotine withdrawal manifestations in rats (Alajaji et al., 2013) and attenuated nicotine preference using the CPP paradigm (Philogene-Khalid et al., 2017). To the best of our knowledge, there are only few studies that report the efficacy of CEF pretreatment in reducing nicotine-seeking behavior, preference in CPP, and withdrawal manifestations. However, as we mentioned in the cannabis section above, these studies used nicotine and not nicotine in cigarettes, and it is difficult to know whether CEF impacts nicotine cigarette addiction.

## Factors Related to the Efficacy of Ceftriaxone in Preclinical Models

As we navigate through the extensive literature documenting the preclinical efficacy of CEF, several questions come to mind. The current working hypothesis entails that CEF upregulates GLT-1 to offset the increase in synaptic glutamate and prevent neuronal toxicity and this glutamatergic fine-tuning leads to attenuation of altered behaviors. In order to fit this model, we may need to explore the temporal relationship among induction of hyperglutamatergic state, CEF-induced GLT-1-upregulation, and elicited behavioral effects.

### Initiation of Ceftriaxone Before the Induction or Early During Hyperglutamatergic State

To the best of our knowledge, almost all studies that tested the efficacy of CEF in neurological disorders have administered CEF prior to the induction or early in the course of the disease models. The small number of studies that used post-treatment administration had negative result outcomes (Chu et al., 2007). (Chu et al., 2007) showed that post-treatment with CEF did not affect the level of infarction volume in rats. Similarly, earl yCEF treatment (from the day of immunization) improved the motor outcome significantly better than late administration (from the onset of motor manifestations) of an MOG peptide immunization mouse model of MS (Melzer et al., 2008). Furthermore, in genetic animal models of cognitive impairment such as the APP/PS1 mouse model of Alzheimer’s disease, CEF improved cognitive impairment if administered in the early stage (Fan et al., 2018).

A similar argument has been made by Kong et al. (2012): adding vitamin E early in the course of ALS in an SOD1 model (at 30 days) to CEF and minocycline (both started at 90 days) improves survival significantly. Along the same line, Keller et al. (2011) have shown that an SOD1 mutant mouse model of ALS treated with minocycline late at 90 days or at 105 days show no significant survival difference compared to control group (144 and 141 days vs. 137 days). However, minocycline-treated animals early at 75 days show better survival compared to control group (147 days vs. 138 days). Therefore, the question of whether early CEF treatment (at time of diagnosis) or pretreatment (high-risk individuals) could attenuate ALS manifestations remains to be answered.

In substance use disorder models, however, the concept of initiating CEF treatment before initiating the hyperglutamatergic state seems less clear. For example, pretreatment with CEF for 5 days prior to first cocaine exposure did not affect the acquisition of cocaine self-administration, while CEF administration after weeks or months of establishing consistent high-ethanol or nicotine consumption patterns shows efficacy in reducing several behavioral manifestations including daily voluntary ethanol drinking or nicotine-seeking behavior (Sari et al., 2011, 2013a,b; Lee et al., 2013; Rao and Sari, 2014a; Das et al., 2015; Rao et al., 2015b; Alasmari et al., 2017; Stennett et al., 2017). This apparent inconsistency could be attributed to the complexity of behavioral disorders, specifically drug addiction, in comparison to structural or motor neurological diseases.

### The Temporal Relationship Between the Upregulation of GLT-1 and the Behavioral Effects of Ceftriaxone

Behavioral effects of CEF have been elicited within 4-7 days in the majority of studies with few exceptions: first acute hyperglutamatergic states such as alcohol withdrawal (Abulseoud et al., 2014), hypoxic-ischemic encephalopathy (Lai et al., 2011), and PTZ-induced seizures (Hussein et al., 2016). Here, CEF works within the first few (Tsai et al., 1998; Campos et al., 2012; Stephens et al., 2014) days. Second, chronic hyperglutamatergic conditions such as cognitive impairment or depression require longer treatment durations. For example, 2 weeks of CEF administration was needed to show an improvement in cognitive function and memory in a PD rat model (Ho et al., 2014), or to reduce depression-like behaviors (i.e., immobility in the forced swim and tail suspension tests) (Rappeneau et al., 2016). Similarly, in a rat model with DLBs, CEF was administered for 4 weeks to elicit an improvement in cognitive function (Ho et al., 2019), and in an 3xTg-Alzheimer’s disease mouse model (Zumkehr et al., 2015). In addition, attenuation of the accumulation of pathological tau in the hippocampus was observed after 8 weeks of CEF treatment in the 3xTg-Alzheimer’s disease mouse model (Zumkehr et al., 2015).

Numerous studies have shown that CEF upregulates GLT-1 mRNA, protein expression, and activity measured as early as 2 (Rao et al., 2015b), 5 (Ackerman et al., 2015; Hu et al., 2015; Sari et al., 2016; Kang et al., 2018), or 7 days post CEF treatment (Abulseoud et al., 2012; Goodrich et al., 2013; Hu et al., 2015). The upregulation of GLT-1 has been suggested to induce the behavioral effects of CEF. However, GLT-1 upregulation is not the sole mechanism.

### The Effect of Pharmacokinetics and Pharmacodynamics Changes on Ceftriaxone Efficacy

For CEF to upregulate GLT1, it needs to achieve a brain tissue concentration of 10 to 100 μmol/L in a tissue culture (Thone-Reineke et al., 2008) or 1 μmol/L in the CSF (Zhao et al., 2014). Incubation of primary astrocytes with CEF at different doses showed significant increase in Glu uptake and GLT1 protein expression at both 10 and 100μmol/L after 2 days of incubation as compared to vehicle (NaCl) (Thone-Reineke et al., 2008). Ceftriaxone administered by continuous intravenous (IV) infusion at 18 mg/h resulted in striatal and CSF CEF concentrations of 0.8 ± 0.17 and 0.7 ± 0.15 μg/ml, respectively (Granero et al., 1995). Similar results were reported in a large multicenter ALS trial. CSF CEF concentrations were measured in a cohort of subjects (*n* = 66) and found to be maintained above the target threshold of 1 μmol/l (0.55 μg/ml) (Zhao et al., 2014). However, several factors affect the ability of CEF in achieving this brain tissue concentration such as expression or inhibition of P-glycoprotein (P-gp). P-gp is an efflux transporter encoded by the ABCB1 gene, and it is located in endothelial cells of the blood brain barrier (BBB). CEF is a substrate for P-gp and P-gp inhibitors such as cyclosporin A and verapamil may enhance the brain concentration of CFF (Shan et al., 2022). Drugs of abuse also affect P-gp expression. We have recently shown that prolonged exposure to ethanol and cocaine alters the expression of P-gp mRNA and protein levels in the NAc and mPFC in alcohol P rats (Hammad et al., 2019). Furthermore, human P-gp was significantly inhibited in a concentration-dependent manner by buprenorphine, methadone, and THC (Tournier et al., 2010). As such, higher brain CEF concentration is expected in animal models of dependence during the drug administration phase compared to the withdrawal or the abstinence phase. Moreover, brain CEF concentration in clinical trials may be influenced by several other factors such as age (Balant et al., 1985; Wang et al., 2020), route of administration (Borner et al., 1985; Albarellos et al., 2007), frequency of dosing (Leegwater et al., 2020), and presence of comorbid medical conditions that could impact clearance or protein-binding state.

In adults, 90-95% of CEF is bound to plasma protein and only a free CEF fraction is pharmacologically active (Hayton and Stoeckel, 1986; Kovar et al., 1997). The free CEF fraction was found to be higher in ICU patients compared to healthy volunteers (Schleibinger et al., 2015). However, in patients with critical conditions, CEF clearance is also increased by 100% and volume of distribution by 90%, leading to variable and, in certain cases, suboptimal plasma CEF concentrations (Joynt et al., 2001). Since protein-binding is saturable, continuous CEF infusion was found to be more effective than intermittent (q12 h or q 24 h) infusion in patients with critical conditions (Leegwater et al., 2020). Similarly, the route of CEF administration may also impact plasma and CSF concentrations and, hence, its efficacy in upregulating GLT1. Peak serum concentration after intramuscular and subcutaneous CEF administrations was not significantly different in cats (54.4 ± 12.9 and 42.35 ± 17.6 μg/ml, respectively) (Albarellos et al., 2007). However, in healthy volunteers, intravenous administration resulted in higher peak plasma CEF concentration [0 min after 2 g intravenous = 258 ± 40 mg/l) than subcutaneous route (37.1 ± 5.6 mg/L after 138 ± 49 min following 2 g subcutaneous) (Borner et al., 1985).

Overall, future clinical trial design should take these factors into consideration, specifically concomitant medications that could facilitate or impede CEF penetration into brain tissue.

### Ceftriaxone Upregulates GLT-1 by Protein Phosphorylation, Thermodynamic Factors, and Intracellular Trafficking

Since NF*k*B, Akt, and other signaling pathways are involved in the modulation of GLT-1 expression (Rao et al., 2015b), CEF works by enhancing the phosphorylation of Akt and NFκB in key brain regions such as the NAc and PFC (Alhaddad et al., 2020). Moreover, CEF increases BDNF and TrkB expression (Thöne-Reineke et al., 2008). It is important to note that studies found that CEF and other β-lactam antibiotics upregulated GLT-1 and xCT expression in the NAc and PFC and reduced ethanol-drinking behavior, and that the effects were associated with alterations of phosphoAKT (pAKT), nuclear factor kappa B (NF*K*B), and an inhibitor of kappa B (IκBα) (Rao et al., 2015a,b). For instance, CEF treatment for 2 and 5 days increased the expression of pAKT and NF*K*B and reduced the expression of IκBα in the NAc and PFC of P rats exposed to ethanol drinking for 5 weeks (Rao et al., 2015b). These signaling pathways are suggested to be part of some of the main mechanistic pathways involved in the upregulatory effects of CEF on GLT-1expression in mesocorticolimbic brain regions. Importantly, 5-week ethanol drinking reduced the expression of pAKT and NF*K*B and increased IκBα expression in the PFC of male P rats (Alhaddad et al., 2014b). This suggests that CEF restores these signaling pathways and consequently reduces ethanol-drinking behavior. There might be other signaling pathways involving CEF-induced upregulation of GLT-1. Studies are warranted to investigate other potential signaling pathways involving CEF effects on the upregulation of GLT-1.

## Other Pharmacological Actions of Ceftriaxone in the Central Nervous System

Ceftriaxone normalizes xCT expression in the NAc and hippocampus (Sari et al., 2009; Knackstedt et al., 2010; Althobaiti et al., 2016; Alshehri et al., 2018) and activates mGlu 2/3 receptors in NAc core (Logan et al., 2020). It also enhances the activity of the GS enzyme (Verma et al., 2010; Fan et al., 2018), decreases microglial activation (Ho et al., 2014), reduces the production of pro-inflammatory mediators such as interleukin 17 and interferon gamma, and attenuates the proliferation of T cells (Melzer et al., 2008). Furthermore, CEF increases neurogenesis (Ho et al., 2019) and improves neuronal integrity through different mechanisms including enhancement of content and activity of antioxidants such as GSH and catalase, respectively (Hussein et al., 2016). These effects are observed in dopaminergic neurons (Ho et al., 2014), where CEF treatment restores dopamine tissue content in the NAc (Althobaiti et al., 2018). In addition, CEF normalizes the firing of LHb neurons (Kang et al., 2018).

### Upregulation of Water Channel Aquaporin-4

It has been found that Aquaporin-4 (AQP-4) is involved in learning and memory, expression of GLT-1, and synaptic plasticity (Li et al., 2012; Szu and Binder, 2016). Moreover, chronic CEF exposure has been reported to improve hippocampal memory and synaptic plasticity in AQP-4 knockout mice by upregulation of GLT-1 expression (Yang et al., 2013).

### Upregulation of Equilibrative Nucleoside Transporter Type 1

In addition to GLT-1, studies from Sari’s laboratory found that CEF also upregulated Equilibrative Nucleoside Transporter Type 1 (ENT1) in mesocorticolimbic brain regions (Sari et al., 2013b). ENT1 has been found to regulate ethanol-drinking behavior through NMDAR signaling pathways (Choi et al., 2004; Nam et al., 2011). Sari’s laboratory previously found that ethanol drinking for 5 weeks increased the activity of ENT1 in NAc shell and NAc core (Sari et al., 2013b). Furthermore, CEF treatment (100 mg/kg IP) for 5 days reduced ethanol-drinking behavior starting from day 2 to day 5 of drug treatment. It is important to note that 3 days after last CEF injection, ENT1 activity has been found to be decreased in both NAc shell and NAc core. This indicates that CEF was effective in restoring ENT1 activity in the mesocorticolimbic system following chronic exposure to ethanol.

### Attenuation of Pro-inflammatory Cytokines

Studies have suggested that the neuro-inflammation associated with multiple sclerosis might lead to high level of extracellular glutamate concentrations in part by reduction in the expression of GLT-1, glutamine synthetase activity, and glutamate dehydrogenase activity (Werner et al., 2001). This suggests that pro-inflammatory cytokines (tumor necrosis factor-alpha, TNF-α, IFNγ, and IL) might be involved in dysregulation of the glutamatergic system. It is important to note that TNF-α increased glutaminase mRNA expression and stimulated the release of glutamate, which might cause neuroexcitotoxicity (Takeuchi et al., 2006). Ceftriaxone was effective in reducing the production of these cytokines in the brain (Chu et al., 2007; Melzer et al., 2008).

It has been found that pre-treatment with CEF (200 mg/kg IP) for 5 days reduced the ischemia-increased mRNA expression of pro-inflammatory cytokines including Fas, FasL,IL-6, and TNF-α (Chu et al., 2007). In addition, attenuation in pro-inflammatory cytokines (IL-17 and IFNγ) concentrations has been observed in dendritic cells incubated with myelin oligodendrocyte glycoprotein following exposure to 500 mM of CEF for 3 days (Melzer et al., 2008). These data were further supported by a study that reported CEF (200 mg/kg IV) improved traumatic brain injury-induced cognitive dysfunction and cerebral edema in part by reduction in the concentrations of pro-inflammatory mediators (IFNγ, TNF-α, and IL-1β) in the brain (Wei et al., 2012). In addition, CEF was suggested to bind to these mediators directly to reduce their activities (Brooks et al., 2001, 2003).

### Reduction of Oxidative Stress

Ceftriaxone has been found to attenuate oxidative stress, consequently leading to neuroprotection against over-activation of certain glutamate receptors, which may lead to excitotoxicity and, consequently, neuronal injury (Amin et al., 2014). Reactive oxygen species (ROS) have been found in mesocorticolimbic brain regions undergoing neurodegeneration in a pre-clinical neurological model [for review refer to Zuo and Motherwell (2013); Huang et al. (2016); Manoharan et al. (2016)]. Although superoxide dismutase enzyme (an antioxidant enzyme) activity was not changed following treatment with CEF (Rothstein et al., 2005), apoptosis and oxidative stress were attenuated after 3 or 7 days of CEF treatments (200 mg/kg IP) (Amin et al., 2014). Oxidative stress has been found to affect the activity of glutamate uptake in astrocytes (Hayashi et al., 2002). Moreover, it has been found that inhibition of glutamate transport function generated oxidative stress radicals, which reduced the ability of the hippocampus to produce antioxidant effects (Nagatomo et al., 2007). Furthermore, it has been reported that treatment with CEF (200 mg/kg IP) ameliorated the memory impairment that was caused by hypobaric hypoxia, and this effect was associated in part by upregulation of GLT-1 in rats (Hota et al., 2008). Importantly, 14-day hypobaric hypoxia induced an oxidative stress characterized by increase in hippocampal LDH enzyme activity, decrease in level of reduced glutathione, and increase in lipid peroxidation, and these effects were attenuated by CEF treatment. This study showed that CEF restored GLT-1 levels in the hippocampus in this hypobaric hypoxia. These findings suggest that CEF has neuroprotective effects indirectly in part by upregulating glutamatergic transporters, and consequently, attenuating the hyperglutamatergic state and stimulating glutathione pathways.

## Clinical Studies Testing the Efficacy of Ceftriaxone

Despite the rich and promising preclinical data showing the efficacy of CEF in attenuating disease manifestations in various neuropsychiatric disorders, to the best of our knowledge, there are less studies on clinical trials that tested the efficacy of CEF. Indeed, the efficacy of CEF in attenuating the progression of motor manifestations was shown in patients with ALS (Cudkowicz et al., 2014), and another study tested its efficacy in reducing post nerve decompressive surgery pain (Macaluso et al., 2013). The search at the clinical trials registration website^1^ shows four more proposed studies: (1) A Glutamate Transporter GLT-1 in the Treatment of Bipolar Disorder (ID# 2007/NCT00512616, sponsored by NIMH, withdrawn), (2) CEF in the Management of Bipolar Depression (ID#2007/NCT00566111, sponsored by Yale, terminated), (3) A Placebo-Controlled Efficacy Study for CEF for Refractory Psychosis (ID#2008/NCT00591318, sponsored by Research Foundation for Mental Hygiene, unknown status), and (4) Efficacy and Safety of CEF for Mild to Moderate Parkinson’s Dementia (ID# 2018/NCT03413384, sponsored by BrainX Corp. in Taiwan, recruiting). The reasons these trials were withdrawn or terminated are not clear.

To the best of our knowledge, only one clinical trial tested the efficacy of CEF in attenuating functional decline [measured by Amyotrophic Lateral Sclerosis Functional Rating Scale-Revised, ALSFRS-R] and improvement of survival in patients with ALS (Cudkowicz et al., 2014). This large-scale, multi-site, double-blind, randomized, placebo-controlled study had three stages; one and two examined the pharmacokinetics and safety of CEF in patients with ALS, and stage 3 included 66 participants from stages 1 and 2 and 448 new participants that were randomly allocated (2:1) to CEF (*n* = 340; 2 or 4 g per day) or placebo (*n* = 173). Among participants randomized to receive CEF, 46% (156/340) remained in the study for 14.2 ± 11.7 months while 41% (71/173) of participants on placebo remained in the study for 12.3 ± 9.2 months. No significant difference between the two groups were found. During stages 1 and 2, functional decline (ALSFRS-R) was 0.51 ± 0.24 (95% CI: 0.0196, 0.9956) units per month slower in participants taking 4 g CEF vs. those taking placebo (*p* = 0.041). In stage 3, there was no significant effect on functional decline (*p* = 0.23). Follow-up for survival was >95% complete; 50% of the participants survived during the study. Log-rank tests showed no significant differences in survival between the two groups (*p* = 0.41).

Despite this negative result, the study provides important safety information for extended IV administration. Aside from gall stones, side effect profile is tolerable. Clearly, it could have been helpful to know the status of GLT-1 and extracellular glutamate concentration before and after CEF treatment. Multimodal imaging by proton (^1^H-MRS) and carbon 13 (^13^C) spectroscopy and developing specific GLT-1 PET ligands are needed to explore glutamate-glutamine cycle dynamics and GLT-1 activity before and after CEF treatment in healthy individuals and in patients with ALS and other neuropsychiatric disorders.

## Conclusion and Closing Remarks

Several studies from our laboratories and others showed the efficacy of CEF in attenuating behavioral manifestations of various hyperglutamatergic disorders in preclinical models. CEF has upregulatory effects on GLT-1 leading to decrease in extracellular glutamate concentration that can be excitotoxic. This may consequently reduce oxidative stress and, possibly, neuroinflammation. Although several studies showed the efficacy of CEF in reducing the hyperglutamatergic state in animal models of drugs of abuse, CEF was less effective in clinical trials involving certain patients with ALS who may suffer from the hyperglutamatergic state (Macaluso et al., 2013; Cudkowicz et al., 2014).

To the best of our knowledge, there is no evidence in humans showing that CEF can modulate the hyperglutamatergic state. We suggest that an MR proton and ^13^C spectroscopy imaging study on human volunteers is needed to determine the changes in glutamate concentrations in key affected brain regions. We also suggest developing specific PET-ligands for GLT-1 to examine the effect of CEF on GLT-1 upregulation in humans. Importantly, we suggest identifying and characterizing new beta-lactams that are devoid of antimicrobial properties to upregulate GLT-1 and attenuate the hyperglutamatergic state. Currently, there is existence of a new beta-lactam, MC-100093 that shows promising results in attenuating cocaine-seeking behavior, and this effect is mediated by upregulation of GLT-1 expression in reward brain regions such as the nucleus accumbens (Knackstedt and Schwendt, 2016).

Overall, CEF may have some potential as a therapeutic agent for many neuropsychiatric disorders, but more preclinical and clinical studies are warranted.

## Author Contributions

OA reviewed the literature on the efficacy of Ceftriaxone in preclinical models of psychiatric and neurological disorders and the clinical trial for ALS and wrote the first draft. FA reviewed the literature on the efficacy of Ceftriaxone on preclinical models of drug addiction and contributed to the manuscript writing. AH reviewed the literature on the efficacy of Ceftriaxone in preclinical models of neurological disorders and contributed to the manuscript writing. YS reviewed the literature on the efficacy of Ceftriaxone in preclinical models of psychiatric and neurological disorders and contributed to the manuscript writing. All authors contributed to the article and approved the submitted version.

## Conflict of Interest

The authors declare that the research was conducted in the absence of any commercial or financial relationships that could be construed as a potential conflict of interest.

## Publisher’s Note

All claims expressed in this article are solely those of the authors and do not necessarily represent those of their affiliated organizations, or those of the publisher, the editors and the reviewers. Any product that may be evaluated in this article, or claim that may be made by its manufacturer, is not guaranteed or endorsed by the publisher.

## Footnotes

1
www.clinicaltrials.gov
